# Characterization at 25 °C of Sodium Hyaluronate in Aqueous Solutions Obtained by Transport Techniques

**DOI:** 10.3390/molecules20045812

**Published:** 2015-04-02

**Authors:** Aleš Mráček, Lenka Gřundělová, Antonín Minařík, Luís M. P. Veríssimo, Marisa C. F. Barros, Ana C. F. Ribeiro

**Affiliations:** 1 Department of Physics and Material Engineering, Faculty of Technology, Tomas Bata University in Zlín, nám. T.G.Masaryka 275, Zlín 762 72, Czech Republic; E-Mail: minarik@ft.utb.cz; 2 Centre of Polymer Systems, Tomas Bata University in Zlín, nám. T.G. Masaryka 5555, Zlín 760 01, Czech Republic, E-Mail: grundelova@cps.utb.cz; 3 Coimbra Chemistry Centre, Department of Chemistry, University of Coimbra, Coimbra 3004-535, Portugal; E-Mails: luisve@gmail.com (L.M.P.V.); marisa.barros@gmail.com (M.C.F.B.)

**Keywords:** diffusion coefficients, transport properties, sodium hyaluronate, Taylor dispersion, Huggins constant, limiting viscosity number

## Abstract

Mutual diffusion coefficients, *D*, were determined for aqueous solutions of sodium hyaluronate (NaHy) at 25 °C and concentrations ranging from 0.00 to 1.00 g·dm^−3^ using the Taylor dispersion technique. From these experimental data, it was possible to estimate some parameters, such as the hydrodynamic radius *R*_h_, and the diffusion coefficient at infinitesimal concentration, *D*^0^, of hyaluronate ion, permitting us to have a better understanding of the structure of these systems of sodium hyaluronate in aqueous solutions. The additional viscosity measurements were done and Huggins constant, *k*_H_, and limiting viscosity number, [η], were computed for interaction NaHy/water and NaHy/NaHy determination.

## 1. Introduction

Sodium hyaluronate or hyaluronan (NaHy) is a polysaccharide with an alternating disaccharide unit (d-glucronic acid and N-acetyl-d-glucosamine) that can be found in various body fluids, tissues and extracellular matrix. This macromolecule was first described by Palmer and Mayer [1]. The behaviours of polymer coils in solutions can play a crucial role in many scientific problems [2]. The highly diluted aqueous solution of NaHy, its conformation and its characterization were recently published by Gřundělová *et al.*, and Verissimo *et al.* [3,4]. Hydrogen bonds acting mostly between water and polysaccharide molecules influence the conformation of NaHy in solutions. The NaHy molecule contains hydrophobic domains in its backbone and hydrophilic groups in its side-branches [5]. The molecules of NaHy can create tertiary structures with itself due to bonding between the hydrophobic patches forming a two-fold helix conformation in water [6,7]. The aforementioned characteristics of NaHy can certainly affect the transport properties of this carbohydrate polymer in water solution.

The transport properties data of these polyelectrolytes in aqueous solutions are of great interest not only for fundamental purposes, but also for many technical fields such as biomedical and pharmaceutical applications. Despite work already done [4,8,9,10], the transport behavior of these systems is still poorly understood. As far as the authors know, only some data on mutual diffusion coefficients of NaHy (relevant for “*in vivo*” pharmaceutical applications) have been published [4]. 

In the present work, the inter-diffusion coefficients and viscosities of aqueous NaHy (from different sources), over the concentration range from 0.01 to 1.0 g·dm^−3^ at 25 °C were measured using a Taylor technique [11,12,13,14,15,16,17,18,19,20,21,22]. From these experimental results, the hydrodynamic radius of the unity of NaHy, *R*_h_, by using Stokes-Einstein equation [11], diffusion coefficient at infinite dilution *D*^0^ and the thermodynamic factors, *F*_T_, by using the Onsager and Fuoss and Gordon equations [23,24] have been estimated, thus allowing us to have a better understanding of the thermodynamics of these amino acids in aqueous solutions.

The effect of the viscosity of the medium on the estimated hydrodynamic radius and also on the behaviour diffusion is also discussed. The dilute solution viscometry data (Huggins constant, *k*_H_, and limiting viscosity number, [*η*]) are very useful for complementary information to the diffusion behaviours of polymers [25], which reflect interactions in binary solutions. Mainly, the Huggins coefficient, *k*_H_, depends on molecular weight and the strength of the polymer-solvent and polymer-polymer interactions. The lower values of *k*_H_ (down to 0.55) are caused polymer-solvent interactions in good solvents at higher molecular weights. However, the higher values of *k*_H_ (between 0.8 and 1) indicate polymer-polymer interactions in poor solvents [25].

## 2. Results and Discussion

### 2.1. Viscosity Data

Results from viscosity measurements in terms of limiting viscosity number and Huggins coefficient *k*_H_ are presented in Table 1. Scott *et al.* [6,26] have shown that generally the hydrophilic polymer chain of NaHy also has hydrophobic microdomains along the chain backbone and the water may always not seem a good solvent. This fact is represented by the Huggins number, *k*_H_ = 0.88 (Table 1), because values up to 0.8 are interpreted as corresponding to bad solvents [25]. Although, this result may appear as unreasonable, because water is generally considered a good solvent for hyaluronan, the conformation parameters of such polymer (*k*_H_) can be, in principle, influenced by additional factors: chain stiffness, interaction between the flexible and stiff segments or intermolecular (in one chain) hydrodynamic interactions of each of the part with the solvent [5,6,7] and, finally, purity of the polymer [27]. Thus, hydrophobic domains of hyaluronan can play a more significant role than hydrophilic interactions, especially for high molecular weights (the molecular weights of the samples used for our experiments was relatively high, *M*_w_=1.8–2.1 MDa). On the other hand, the viscosity data can be influenced by the polydispersity of the polymer or thermal conditions of preparations [28,29,30].

**Table 1:** Values of relative, specific, reduced viscosity, LVN and Huggins parameter.

***c*_NaHy_ [g·dm^−3^]**	1	0.7	0.5	0.25	0.1
**_rel_**	56.4	29.3	13.4	6.2	2.3
**η_spec_**	55.4	28.3	12.4	5.2	1.3
**η_red_ [dm^3^·g^−1^]**	55.4	40.5	24.8	20.8	13.3
**[η] [dm^3^·g^−1^]**	7.27				
***k*_H_**	0.88				

### 2.2. Concentration Dependence of Mutual Diffusion Coefficient, D, at Infinitesimal and Finite Concentrations

Table 2 gives the average *D* value at infinitesimal concentration for each injection solution determined from four to five profiles generated by different injecting samples in water. *D*^0^ is obtained by extrapolated values obtained from the *D* least-squares for total number of injections (that is, *D*^0^ = 1.333 × 10^−9^ m^2^·s^−1^).

**Table 2:** Mutual diffusion coefficients, *D*, of NaHy aqueous solutions at different concentrations and the respective standard deviations, *S*_D_, obtained from the Taylor technique by using pure water as carrier stream, at *T* = 25 °C and at atmospheric pressure.

*c*/(g·dm^−3^)	*D* ± *S*_D_^(a)^/(10^−9^ m^2^·s^−1^)
1.000 2.000 3.000 4.000 5.000	1.232 ± 0.040 1.110 ± 0.030 1.008 ± 0.037 0.901 ± 0.038 0.786 ± 0.040
	*D*^0^ = 1.333 × 10^−9^ m^2^·s^−1 (b)^

^(a)^ *D* is the mean diffusion coefficient value from 4–6 experiments and *S*_D_ is the standard deviation of that mean, for hyaluronic acid sodium salt supplied by Contripo Biotech s.r.o gal. u(*c*) = 0.0001 g·dm^−3^ u(*D*) = 0.02 × 10^−9^ m^2^·s^−1^; u(*T*) = 0.01 °C; ^(b)^ Extrapolated value obtained from the *D* least-squares for the total number of injections, where *D***/**(10^−9^ m^2^·s^−1^) = 1.333–0.109*c* (*R*^2^ = 0.997).

Table 3 gives the average *D* value for finite concentrations at six carrier solutions, determined from four to five profiles generated by injecting samples in those solutions. Good reproducibility was, in general, observed, within ±2%. 

The concentration dependence of the measured diffusion coefficients can be represented by the polynomial equation:

*D/*(10^−9^ m^2^·s^−1^) = 1.3176 − 6.194 *c*^1/2^ + 12.747*c* (c ≤ 0.1 g·dm^−^^3^) (*R*^2^ = 0.990) (1)

Permitting us to calculate values of diffusion coefficients at specified concentrations within the range of the experimental data shown in the Table 3. The goodness of the fit (obtained with a confidence interval of 98%) can be assessed by the excellent correlation coefficients, *R^2^* and the low percentage of standard deviation (<1%).

**Table 3:** Mutual diffusion coefficients, *D*, of NaHy in aqueous solutions at different concentrations, c, and the respective standard deviations, *S*_D_, obtained by the Taylor technique (by using carrier stream solutions of different concentrations) at *T* = 25 °C and at atmospheric pressure.

*c*/(g·dm^−3^)	*D* ± *S*_D_^(a)^/(10^−9^ m^2^·s^−1^)
0.010 0.025 0.050 0.100 0.250 1.000	0.780 ± 0.020 0.670 ± 0.018 0.602 ± 0.021 0.619 ± 0.010 0.560 ± 0.015 0.500 ± 0.018

^(a)^ *D* is the mean diffusion coefficient value from 4–6 experiments and *S*_D_ is the standard deviation of that mean, for hyaluronic acid sodium salt supplied by Contripo Biotech s.r.o gal. u(*D*) = 0.02 × 10^−9^ m^2^·s^−1^; u(*T*) = 0.01 °C.

### 2.3. Influence of the Kinetic and the Thermodynamic Factors on the Behaviour Diffusion of NaHy in Aqueous Solutions at Finite Concentrations

The analysis of the diffusion behaviour of this aqueous system (sodium hyaluronate) can be done on the basis of the Onsager-Fuoss theory (Equation (2)) [23,24], suggesting that *D* is a product of both a kinetic, *F*_M_ (or molar mobility coefficient of a diffusing substance) and a thermodynamic factors, *F*_T_ (*F*_T_ = *c*∂*μ*/∂*c*), where μ represents the chemical potential of the solute. Two different effects can control the diffusion process: the ionic mobility and the gradient of the free energy:

*D*_OF_ = *F*_M_ × *F*_T_ (2)

where:

(3) $$F_{\text{M}}=\bar{M}\;\left(\frac{\left|Z_{\text{c}}\right|+\left|Z_{\text{a}}\right|}{\left|Z_{\text{c}}Z_{\text{a}}\right|}\right)\frac{\text{R }T}{c}=\left(D^{0}+{\text{Δ}}_{1}\right)$$

and:

(4) $$F_{\text{T}}=\left(1+c\frac{\partial \;ln\;y_{\pm }}{\partial c}\right)$$

being *c* the concentration in mol.m^−3^, $y_{\pm }$ the mean molar activity coefficient of the solute, the other symbols having their usual meaning, and $\bar{M}$, in mol^2^·s·m^−3^·kg^−1^, is given by:

(5) $$\bar{M}=\frac{1}{{\text{N}}_{\text{A}}^{2}{\text{e}}_{0}^{2}}\left(\frac{\lambda_{\text{c}}^{0}\;\lambda_{\text{a}}^{0}}{v_{\text{a}}\left|z_{\text{a}}\right|\lambda_{\text{c}}^{0}+v_{\text{c}}\left|z_{\text{c}}\right|\lambda_{\text{a}}^{0}}\right)\;c+\bar{\text{Δ}M’}$$

where the first-order electrophoretic term, is given by:

(6) $$\Delta \bar{M’}=-\frac{c}{{\text{N}}_{\text{A}}}\frac{{\left(\left|z_{\text{a}}\right|\lambda_{\text{c}}^{0}-\left|z_{\text{c}}\right|\lambda_{\text{a}}^{0}\right)}^{2}}{{\left(\left|z_{\text{c}}\right|\nu_{\text{c}}\lambda_{\text{a}}^{0}+\left|z_{\text{a}}\right|\nu_{\text{a}}\lambda_{\text{c}}^{0}\right)}^{2}}\frac{\nu_{\text{c}}\nu_{\text{a}}}{\nu_{\text{c}}+\nu_{\text{a}}}\frac{k}{{6\pi \;\eta }_{0}\left(1+ka\right)}$$

where η_0_ is the viscosity of water in Pa.s, N_A_ is the Avogadro’s constant, e_0_ is the proton charge in coulombs, *ν*_c_ and *ν*_a_ are the stoichiometric coefficients, *k* is the “reciprocal average radius of ionic atmosphere”, in m^−1^ (see e.g., [24], *a* is the mean distance of closest approach of ions in m (we used *a* = 5.0 × 10^−10^ m) and the other symbols have their usual meaning. From our measurements of diffusion coefficients, *D*, and considering Equation (2), we have estimated the thermodynamic factor values for different concentrations (Table 4).

**Table 4:** Mobility factor, *F*_M_, and thermodynamic factor, *F*_T_, of NaHy alculated from our experimental *D* values and from Equations (3) to (7) at *T* = 25 °C and at atmospheric pressure.

*C* (g·dm^−3^)	Δ_1_/10^−9^ m^2^·s^−1^ M = 1.8 × 10^6^ Da ^(a)^	*F*_M_ 10^−9^ m^2^·s^−1^ ^(b)^	*F*_T_ 10^−9^ m^2^·s^−1^ ^(c)^	*F’*_T_ 10^−9^ m^2^·s^−1^ ^(d)^
0.000 0.010 0.025 0.050 0.100 0.250 1.000	0 5.00 × 10^−4^ 5.01 × 10^−4^ 5.01 × 10^−4^ 5.01 × 10^−4^ 5.02 × 10^−4^ 5.03 × 10^−4^	1.333 1.333 1.333 1.333 1.333 1.333 1.333	1.000 0.585 0.503 0.452 0.464 0.420 0.375	1.000 0.877 0.860 0.812 ^(f)^ 1.087 2.605 21.15

^(a)^ Δ_1_ represents the electrophoretic correction for the molecular mass of the hyaluronic acid sodium salt *M* = 1.8 MDa. This parameter is estimated by using Equations (4), (6) and (7); ^(b)^ *F*_M_ = (*D*^0^ + Δ_1_), where *D*^0^ is the diffusion coefficient at infinitesimal concentration; ^(c)^ *F*_T_ = *D*_exp_ / *F*_M_; ^(d)^ *F’*_T_ = *D*_exp_ η*_r_*/*F*_M_, being η*_r_* the relative viscosity (Equation (7)) of this work [11]; ^(f)^ *F’*_T_ = *D*_exp_ η*_r_*/*F*_M_, being η*_r_* = 1.797 estimated by interpolation using the experimental data (see Table 2).

The values for Δ_1_, shown in Table 4, are very small (only contributing 0.1%, approximately, to the decrease of *D^0^*) and, consequently, *F*_M_ is almost constant for the concentration range studied. 

The decrease of the diffusion coefficients, *D*, and also of the gradient of the free energy with concentration, *F*_T_, (fourth column in Table 3) leads us to conclude that this behavior observed for the sodium hyaluronate in aqueous solutions at *T* = 298.15 K appears to be affected by the presence of aggregated species (this fact is confirmed by molecular mechanic calculations), which have a lower mobility than the alginate monomers due to their size. Considering our experimental conditions (*i.e.*, dilute solutions) and assuming that parameters such as viscosity, dielectric constant and hydration, (factors not taken into account by this model) do not change with concentration, we can conclude that the variation in *D* is mainly due to the variation of *F*_T_ (attributed to the non-ideality in thermodynamic behaviour) and, in a lesser amount, to the electrophoretic effect in the mobility factor, *F*_M_ (Table 4). 

### 2.4. Influence of the Viscosity Factor on the Behaviour Diffusion of Hyaluronate in Aqueous Solutions at Finite Concentrations

The diffusion of this polyelectrolyte in aqueous solutions may also depend on the viscosity change in the solution (negligible in dilute solution, but becoming important when the concentration increases) and, consequently, the estimated values of *F*_T_ in the absence of this viscosity-effect (Table 5) may show large deviations from the real values. Thus, on the basis of Gordon’s equation [23,24]:

(7) $$D_{\text{G}}=D_{\text{OF}}\left(\frac{\eta_{0}}{\eta }\right)$$

where $\eta_{0}$ and η represent the viscosity of water and the solution, respectively, and *D*_OF_ is the diffusion coefficient obtained by the Onsager-Fuoss Equations (2)–(6), we have estimated a new *F’*_T_ values (last column in Table 4) as equal to:

(8) $${F’}_{\text{T}}=F_{\text{T}}\left(\frac{\eta }{\eta_{0}}\right)$$

These *F*’_T_ values are, obviously, higher than those of *F*_T_ calculated from Equation (8) becoming the discrepancies more significant when the concentration increases. This fact of increasing of the thermodynamic factor makes the contribution of the *F*_M_ factor to be even smaller when the concentration of sodium hyaluronate becomes higher. The anomalous values of *F*_T_ for *c* = 0.250 g·dm^‑3^ and *c* = 1 g·dm^−3^ may be explained having in mind the limitations of the Gordon’model, being in general valid for dilute solutions (*i.e.*, *c* ≤ 0.01 g·L^−1^, Table 4).

**Table 5:** Comparison between the measured and theoretical mutual diffusion coefficients, *D*_G_ and *D*_G’_, of NaHy at *T* = 25 °C in aqueous solutions at finite concentrations, *c*.

c/(g·dm^−3^)	D_G_^(a)^/(10^−9^ m^2^·s^−1^)	ΔD/D% ^(b)^	D_G’_^(c)^/(10^−9^ m^2^·s^−1^)	ΔD/D% ^(d)^
0.010 0.025 0.050 0.100 0.250 1.000	1.141 0.949 0.742 0.580 0.215 0.024	−32 −29 −19 +8.6 +160 +1983	1.001 0.816 0.602 0.630 0.560 0.650	−22 −18 0.0 −1.7 0.0 −0.4

^(a)^ *D*_G_ represent the diffusion coefficient estimated by Gordon equation [23] (*D*_G_ = *D*_OF_ *F*_T_/η*_r_*), using our values of viscosity and the values of *F*_T_ indicated in Table 4; ^(b)^ Δ*D/D%* represent the deviations between our diffusion coefficients and the values calculated by Gordon equation, using values of *F*_T_; ^(c)^ *D*_G_’ represent the diffusion coefficient estimated by Gordon equation [23] (*D*_G_ = *D*_OF_ *F’*_T_/η*_r_*), using our values of viscosity and the values of *F’*_T_ indicated in Table 4; ^(d)^ *∆D/D%* represent the deviations between our diffusion coefficients and the values calculated by Gordon equation, using values of *F’*_T_.

This treatment has been so far phenomenological, and therefore, the dependence of *D* and *F*_T_ on factors such as the nature of the ion and different types of interactions cannot be theoretically understood. However, we may interpret that behaviour on the basis of weak interactions water/sodium hyaluronate. In fact, assuming the possibility that some water molecules are tightly bound to certain ions, they cannot be effective in dissolving further ions added. Therefore, as the concentration of sodium hyaluronate increases, the amount of effective water decreases, and the activity coefficient increases. The hydration of these ions reduces the amount of free solvent from the present for a given stoichiometric concentration, then the effective concentration increases and the activity coefficient must also increase. In our case, this increasing is not compensated for the decreasing due to interactions sodium cation hyaluronate anion, and of course it is not unreasonable that the activity coefficient and, consequently, the thermodynamic factor should rise above the unity.

### 2.5. Hydrodynamic Radius of the Unit of NaHy

The hydrodynamic radius of the hyaluronate anion can be estimated by using the well known Stokes–Einstein equation (Equation (9)), which assumes that the particles are perfectly spherical and are solely subject to solvent friction. That is:

(9) $$D_{\text{T}}^{0}=\frac{{\text{k}}_{\text{B}}T}{6\pi \eta_{0}R_{\text{h}}}$$

where *k*_B_, *R*_h_ and $D_{\text{T}}^{0}$ represent the Boltzmann constant, hydrodynamic radius of an equivalent spherical particle, *R*h, and its diffusion coefficient at infinitesimal concentration, $D_{\text{T}}^{0}$, (known as tracer diffusion coefficient), respectively. Thus, the Stokes radius of the hyaluronate anion estimated from this equation, which assumes that the particles are perfectly spherical and are solely subject to solvent friction (Table 6) is equal to 0.184 nm, under infinitesimal ionic strength conditions.

**Table 6:** Hydrodynamic radius, *R_h_,* of the unity NaHy at solutions of the different concentrations, *c*, and different viscosities, η, at *T* = 25 °C and at atmospheric pressure.

*c*/(g·dm^−3^)	*R*_h_/nm
0.000 0.010 0.025 0.050 0.100 0.250 1.000	0.184 0.269 0.261 0.266 0.172 0.071 ^(a)^ 0.009 ^(a)^

^(a)^ These values very low may be justified if we consider the limitations of Stokes relation and, consequently, we are not considered in the estimation of the hydrodynamic radius, *R*_h_, of the unity NaHy.

By taking into account the Stokes equation and replacing the water viscosity by the viscosity of the solutions, the values of the effective hydrodynamic radius *R*_h_ of hyaluronate are estimated as a function of the concentration of the solute and collected in Table 6. In general, the significant differences between each one and the infinitesimal value for the effective hydrodynamic radius *R*_h_ (approximately 30% for c ≤ 0.1 g·dm^−3^) come again to support the importance that the viscosity effect has on the properties of the sodium hyaluronate solutions. In addition, as it can be observed, for c ≤ 0.05 g·dm^−3^ the maximum variation observed in these *R*_h_ values, is around 3%. For c > 0.05 g·dm^−3^ the significant differences may be justified if we consider the limitations of Stokes relation. Although this relationship is only approximated (arising from the acceptance that both the solute kinetic species and the solvent are not structured, together with the assumption that viscosity is the only responsible of the diffusivity reduction), it can be used to estimate the radius of the moving species, since alginate ions are large enough when compared with water molecules.

## 3. Experimental Section 

### 3.1. Materials

Sodium hyaluronate (NaHy) samples (*M*_w_ = 1.8–2.1 MDa, Cosmetic quality class, batch number: 060413-2) were kindly provided by Contipro Biotech, Ltd. (Dolní Dobrouč, Czech Republic). For the viscosity measurements, stock solution of NaHy in de-ionized water (resistivity *R* = 18.2 × 10^4^ Ω·m, Direct-Q 3 UV, Millipore, Molsheim, France) was prepared in concentration of 0.1% (w/w, 1 g·L^−1^) by slowly adding polymer to the water solution under continuous stirring, followed by 24-h dissolving at 50 °C. The solutions for the diffusion measurements were prepared in calibrated volumetric flasks using also Milli-Q^®^ water (from A10 Millipore^®^) and were freshly prepared.

### 3.2. Methods

#### 3.2.1. Mutual Diffusion Coefficients, D, Measurements from Taylor Technique

**Figure 1:**
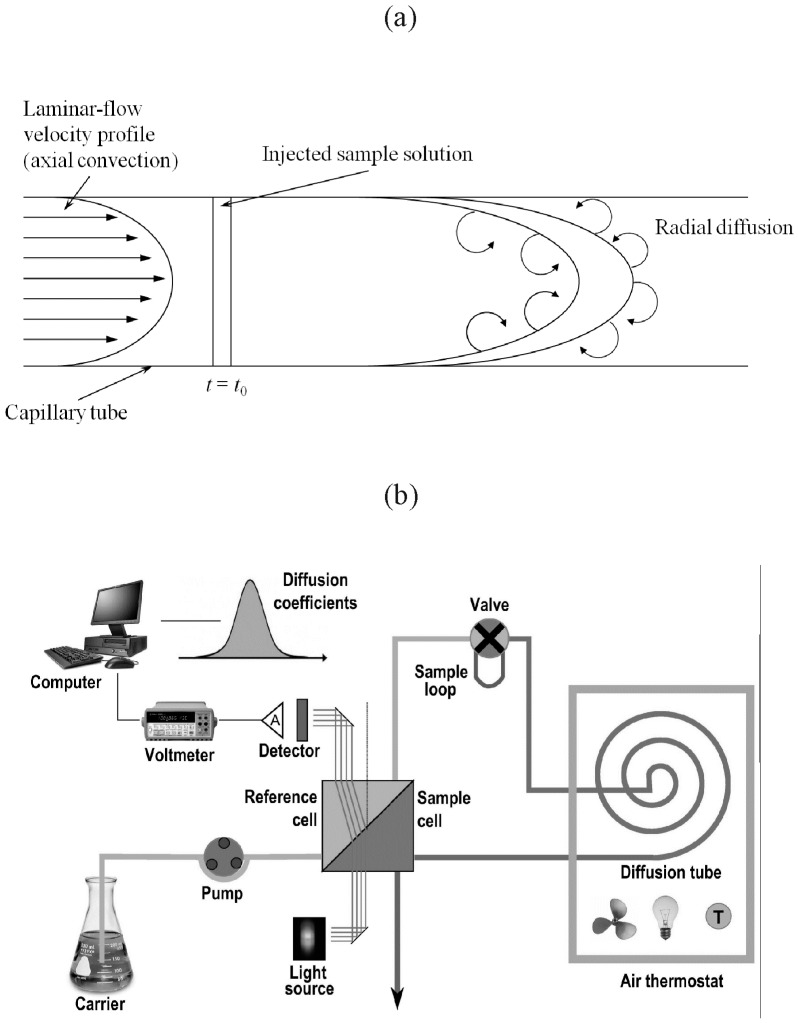
Schematic representation of the Taylor dispersion technique, (**a**) The principle of flow and diffusion in capillary tube; (**b**) The arrangement of the measuring apparatus.

The theory of the Taylor dispersion technique is well described in the literature [11,12,13,14] and consequently the authors only point out some relevant points concerning such method on the experimental determination of binary diffusion coefficients and ternary diffusion coefficients, respectively. Dispersion methods for diffusion measurements are based on the dispersion of small amounts of solution injected into laminar carrier streams of solvent or solution of different composition, flowing through a long capillary tube (Figure 1a,b). The length of the teflon (PTFE) dispersion tube used in the present study was measured directly by stretching the tube in a large hall and using two high quality theodolytes and appropriate mirrors to accurately focus on the tube ends. This technique gave a tube length of 3.2799 (± 0.0001) × 10^3^ cm, in agreement with less-precise check measurements using a good-quality measuring tape. The radius of the tube, 0.05570 (± 0.00003) cm, was calculated from the tube volume obtained by accurately weighing (resolution 0.1 mg) the tube when empty and when filled with distilled water of known density. 

At the start of each run, a 6-port Teflon injection valve (model 5020, Rheodyne, IDEX Health & Science LLC, Oak Harbor, WA 98277, USA) was used to introduce 0.063 cm^3^ of solution into the laminar carrier stream of slightly different composition. A flow rate of 0.17 cm^3^.min^−1^ was maintained by a metering pump (model Minipuls 3, Gilson, Inc., Middleton, WI 53562-0027, USA) to give retention times of about 8 × 10^3^ s. The dispersion tube and the injection valve were kept at 298.15 K and 303.15 K (± 0.01 K) in an air thermostat.

Dispersion of the injected samples was monitored using a differential refractometer (model 2410, Waters Corporation Inc., Milford, MA 01757, USA) at the outlet of the dispersion tube. Detector voltages, *V*(*t*), were measured at accurately timed 5 s intervals with a digital voltmeter (34401 A, Agilent Technologies, Santa Clara, CA, 9505, USA) with an IEEE interface. Binary diffusion coefficients were evaluated by fitting the dispersion equation:

*V*(*t*) = *V*_0_ + *V*_1_*t* + *V*_max_ (*t*_R_/*t*)^1/2^ exp[–12*D*(*t* − *t*_R_)^2^/*r*^2^*t*] (10)

to the detector voltages. The additional fitting parameters were the mean sample retention time *t*_R_, peak height *V*_max_, baseline voltage *V*_0_, and baseline slope *V*_1_.

#### 3.2.2. Viscosity, η, Measurements Obtained from Ubbelohde Viscometer 

The kinematic viscosity measurement on NaHy solutions was carried out in a Ubbelohde capillary viscometer II, Schott (constant K = 0.1024 × 10^−3^ m^2^·s^2^, diameter d = 1.13 × 10^−3^ ± 0.01 m). The viscometer was placed into a water-cooled bath (Thermostatic bath Huber CC-130A Visco 3) with the temperature 25.0 ± 0.1 °C. The flow times of NaHy solution were measured for five different concentrations (for stock solution 1 g·dm^−3^ and four dilute solutions of stock: 0.7 g·dm^−3^, 0.5 g·dm^−3^, 0.25 g·dm^−3^ and 0.1 g·dm^−3^). Values of limiting viscosity number (LVN) [η] and Huggins parameter *k*_H_ were then determined from the flow times measured with these polymer solutions and the linear least square regression of the η_sp_/*c* versus *c* dependence was used for the [η] and *k*_H_ calculation (*c* is polymer concentration in g·dm^−3^, η_sp_ is specific viscosity).

## 4. Conclusions 

From mutual diffusion coefficients measurements for aqueous solutions of sodium hyaluronate (NaHy) at 25 °C and concentrations ranging from 0.00 to 1.00 g·dm^−3^, we conclude that the diffusion of this polysaccharide in aqueous solutions is strongly affected by the presence of new different species resulting from various interactions (such as interactions between the flexible and stiff segments or intermolecular hydrodynamic interactions of each of the part with the solvent) and, consequently, to the decreasing of the diffusion coefficients with the increasing of concentration. The effect of those interactions on the diffusion of NaHy, confirmed by analysis of the dependence of diffusion on concentration is mainly due to the variation of *F*_T_ (attributed to the non-ideality in thermodynamic behaviour), and, secondarily, to the electrophoretic effect in the mobility factor, *F*_M_. 

On the other words, considering that *D* is a product of two factors (kinetic and thermodynamic), the mobility of these ions (*i.e*., sodium and hyaluronate ions) in diffusion varies much less with concentration than does their gradient of chemical potential. This difference is due to the fact that in diffusion the ions move in the same direction. Consequently, the presence of interionic effects leads us a small electrophoretic effect. 

Taking in consideration the effect of viscosity on diffusion of this polyelectrolyte in the Gordon equation, we obtain results closer to the experimental data. We can conclude that the behavior of the polyelectrolyte depends on the viscosity change of the solution. In fact, the variation of the viscosity is much greater than the variation of the diffusion coefficient for the same interval of concentrations. 

Diffusion coefficients, together with viscosity data measured for NaHy in aqueous solutions may provide transport data necessary to model the diffusion in pharmaceutical and biological applications.
